# Social cognitive impact of essential tremor

**DOI:** 10.1038/s41598-025-08402-7

**Published:** 2025-07-02

**Authors:** Sarah Melchert, Oliver L. Steiner, Christin Kupper, Hannah Lochmann, Michelle Wyrobnik, Fabian Klostermann

**Affiliations:** 1 Motor and Cognition Group, Department of Neurology, Charité – University Medicine Berlin, Freie Universität Berlin and Humboldt- Universität zu Berlin, Campus Benjamin Franklin (CBF), 12203 Berlin, Germany; 2 Berlin School of Mind and Brain, Humboldt-Universität Zu Berlin, Berlin, Germany; 3 Institute of Psychology, Humboldt-Universität Zu Berlin, Berlin, Germany

**Keywords:** Neurology, Movement disorders, Psychology

## Abstract

Though classified as a movement disorder, essential tremor (ET) goes along with minor cognitive change. This mainly refers to executive functions, thought to be of paramount importance for social cognition, particularly cognitive theory of mind (ToM). Therefore, different ToM and executive tasks were assessed in persons with versus without essential tremor. 21 non-demented patients with essential tremor and 29 healthy controls underwent cognitive screening, different tasks addressing executive functions, and the faux pas recognition test (FPRT) as well as the reading the mind in the eye test (RMET), focusing on cognitive and affective ToM, respectively. Patients performed significantly worse than controls in the verbal fluency and the digit span tests as well as in the FPRT. No significant group differences were identified with respect to RMET performance. The results are compatible with the idea that persons with ET develop subtle cognitive ToM deficits in the context of executive dysfunction. This extends descriptions of the non-motor impact of ET by deficits in social cognition and raises the question whether potential cognitive change of affected patients is sufficiently acknowledged in clinical routine.

## Introduction

Essential tremor (ET) is one of the most frequent movement disorders with a relatively benign course and certain familial clustering. Traditionally, it is considered as a monosymptomatic disorder, occasionally accompanied by mild cerebellar signs. However, several reports described an association of ET with particular personality traits and subtle cognitive changes^1^. For example, in patients with ET refractory to pharmacological treatment abnormally low cognitive task performances were found in the neuropsychological assessment before surgery for Deep Brain Stimulation (DBS)^2,3^. While one could argue that DBS candidates are the most severely affected ET patients so that a selection bias in such findings cannot be excluded, similar results were also raised in three population-based trials investigating persons with ET^4–6^. Further, based on brain imaging ET-related affection of cognitive networks was presumed, given correlations of low task performances with reduced grey matter volume in cerebellar and cortical areas^7^. Finally, in persons with ET, who additionally suffer from mild cognitive impairment, progression to dementia was found faster than suggested by typical conversion rates reported in the literature^8^.

Concerning cognitive symptoms specifically associated with ET, different authors described deficits in tasks imposing relatively high executive demands, e.g., in working memory, attention, concept formation, and verbal fluency tests^7,9^. Based on this, one could assume an impact of the condition also on particular aspects of social cognition. Executive functions are thought to be most relevant for cognitive Theory of Mind (ToM), but less so for its affective dimension^10–14^. Affective ToM is thought to involve mirroring processes for simulating the mental state of another person in one’s own mind, eventually enabling empathetic behavior. By contrast, cognitive ToM refers to inferential and strategic interpretations of other persons’ mindsets, including intentions, motivations, goals, and so on, for example, in order to align one’s own behavior to the actual social conditions. In so doing, it relies on basic executive operations, such as shifting, inhibition, selection, and updating, e.g., if one has to change between the perspectives of different protagonists in an observed scene, filter out input irrelevant to their interaction, or integrate perceived information into the ongoing context^15^.

The question of ToM change may at first sight seem counterintuitive in ET, which is mainly characterized by cerebellar and brainstem pathology^16^. However, social cognitive deficits can indeed be found in conditions without leading cortical affection^17,18^. In this regard, the probably best studied condition is Parkinson’s disease (PD)^19^. Different trials showed that cognitive ToM abnormalities prevail already in early stages, when PD brain pathology remains largely confined to the basal ganglia^20–23^. A possible explanation for this is cortical diaschisis due to subcortical dysfunction^22,24^. Using the example of PD, this would mean that abnormal striatal output disturbs downstream processing in the dorsolateral prefrontal cortex, implied in executive and social cognitive processing. Accordingly, associated ToM and dysexecutive symptoms in PD might be interrelated facets of a frontostriatal network disorder^25^. Interestingly, cognitive abnormalities in ET and PD overlap, e.g., in memory tasks with high executive demands^26^. A reason for this could be that in ET the cerebellar pathology impacts on projection targets, also affected in PD. Specifically, recent functional imaging data from ET patients showed decreased connectivity of the dentate nucleus as the main cerebellar output structure with remote brain structures, among others, the caudate nucleus and prefrontal cortical areas, and this disconnection pattern went along with low cognitive performance^24,27^.

Considering the impact of cerebellar processing on prefrontal functioning, we posit that ToM capacities, particularly in its cognitive domain, are reduced in persons with ET together with abnormalities in executive task performances. To test this assumption, different corresponding task performances were assessed in persons with and without ET and compared between the groups. The study of this topic is important, since social cognition is of paramount relevance for everyday functioning and because ET patients themselves reported changes in various social life aspects^28^, whose interpretation as a reaction to the visible disease stigma might fall short of additional explanations.

## Methods

### Patients

We recruited 21 ET patients from the neurological outpatient clinic of the Charité, Campus Benjamin Franklin. The diagnosis was confirmed by an experienced neurologist, specialized in the field of movement disorders. Symptomatic tremor forms caused by pathologies such as cerebellar lesions, alcohol abuse or neuropathic syndromes were excluded. Further, patients with dementia (for cutoff values see below), substance abuse, or regular administration of centrally active drugs, including primidone for tremor suppression, were also excluded. Participants had to be native German speakers, since all tests were provided in German. The tasks were performed in the same order by all participants, breaks could be taken as desired. Besides, 29 healthy controls were recruited from the pool of accompanying persons of patients. Between the groups, participants were matched for disease-independent sociodemographic factors, i.e., age, sex distribution, and the years of education, which could have influenced the further test results. We did not match the groups with respect to the screening results for depressiveness and cognition, because lowered mood values and cognitive performances are subtle, yet significant non-sensorimotor aspects of ET, so that their alignment would have necessitated the recruitment of an unrepresentative patient group. However, no cases of severe depression were present in the study cohorts. All participants gave written informed consent to the study protocol approved by the ethics committee of the Charité (EA4/165/17).

### Cognitive assessments

The reading the mind in the eye test (RMET) and the faux pas recognition test (FPRT) were used as standard tools to assess affective and cognitive ToM, respectively. For the RMET, participants are presented with 36 images of the eyes’ region showing different emotions and are required to pick the adjective describing the emotional state adequately out of four possible options^29^. Each correct answer accounts for one point, so that the maximally achievable score is 36. Cognitive ToM was assessed by the FPRT^30^. The participants were read 10 stories, in five of which one of the characters acted in a socially inappropriate way, in other words, committed a faux pas. The FPRT comprises 5 tasks; (i) the occurrence of a faux pas had to be generally detected and the person committing the faux pas had to be recognized (detect), (ii) the inappropriate action or statement had to be identified (inappropriateness), (iii) the intention behind the misconduct had to be understood (intention), (iv) assumptions about the cognitive assessment of the situation (belief) and (v) the involved persons’ emotional response to the situation had to be inferred (empathy). For the first task, the faux pas detect, two points could be reached, one for the detection of inappropriate conduct and one for the correct identification of the responsible person; for each of the four remaining correctly performed tasks one point was assigned, leading to a maximum score of 6 points per story. If no faux pas occurred and this was correctly noted, subscores were not built and the participant received 2 points. Per story, two additional control questions addressed the general understanding. Wrong answers to these questions led to exclusion of the respective story.

Existing literature about cognition in persons with ET reported almost unanimously deficits in verbal fluency, which was therefore assessed by the German standard test (Regensburger Wortflüssigkeitstest). The participants had to produce as many words as possible during two minutes in the two task types ‘semantic’ and ‘phonemic’, each to be performed in two conditions^31^. For the semantic part, words from a certain category had to be produced either alternatingly (animals vs. furniture) or non-alternatingly (vegetables); for the phonemic part, words starting with a certain letter had to be named also either alternatingly (g-words and r-words) or non-alternatingly (s-words). Proper names and words derived from the same word stem were not allowed. Furthermore, working memory performance was assessed with the digit-span-test, for which an increasing succession of numbers had to be repeated either in the order in which they were presented (DS-forward), or in reverse order (DS-backwards)^32^. As a cognitive screening test the Parkinson Neuropyschomotoric Dementia Assessment (PANDA)^33,34^ was applied, originally developed for sensitive detection of cognitive abnormalities emerging in the course of PD. As in previous trials involving patient groups with conditions other than PD^17,18^, we used the PANDA, since the present study was part of a larger series of investigations originally implying participants with PD. Principally, the PANDA is constructed similarly to the Montreal Cognitive Assessment. Its total score, ranges from 0 (worst) to 30 (best) and provides an index of the general cognitive status. It comprises subtests in the domains verbal and non-verbal memory, working memory/attention, executive functioning, visuospatial abilities, and language.

### Clinical assessment

With respect to tremor severity, the Tremor Research Group Essential Tremor Rating Scale (TETRAS) was used. It comprises two sub scales summed up to a total score (with a maximum of 112 points, indicating the worst tremor condition), firstly, for the assessment of the tremor impact on activities of daily living, and, secondly, for the documentation of the examined rest and activity tremor intensity as well as its distribution at head, right/left and upper/lower limbs and effect on the voice^35^. Health-related quality of life was determined using the Parkinson’s Disease Questionnaire (PDQ39), substituting the term Parkinson’s disease by the term Essential Tremor. The questionnaire investigates various quality of life aspects, namely activities of daily life, emotional well-being, social support, communication, bodily discomfort, perceived stigma and cognitive impairment, using 39 questions that can be answered with gradings from 0 (absent) to 4 (always present), higher scores indicating worse conditions^36^. For the assessment of depression, fatigue and daytime sleepiness the Hamilton depression scale (> 8 points for mild, > 16 points moderate and > 24 severe depression), the Fatigue Severity Scale (9–63 points, higher results indicating higher severity of fatigue), and the Epworth Sleepiness Scale (0–24 points, higher results indicating more severe daytime sleepiness) were used^37–39^.

### Statistics

All statistical analyses and figures were performed using Python 3.12.4. First, we compared group differences between ET patients and controls using t-tests for normally distributed variables and Mann–Whitney U tests for non-parametric data. To investigate the interaction effects within Verbal Fluency and Digit Span, we employed linear mixed models with random effects for subjects and fixed effects for the task type (verbal fluency: semantic vs. phonemic) and condition (verbal fluency: alternating vs. non-alternating; Digit Span: backward vs. forward).

Lastly, we analyzed associations in the ET and control group using Pearson’s correlation for normally distributed data and Spearman’s rank correlation for data not normally distributed.

## Results

### Group differences

**Fig. 1:**
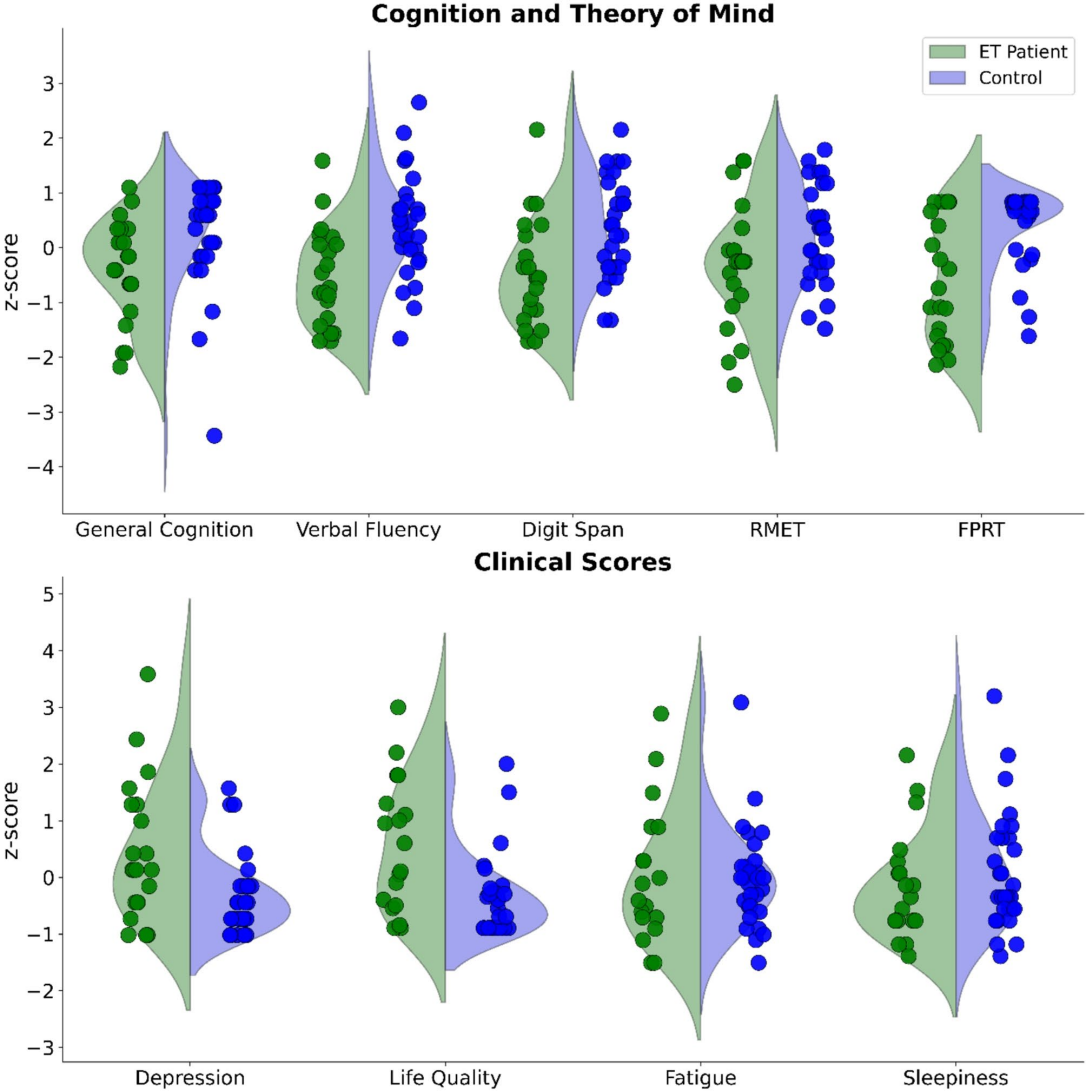
The violin plots compare healthy controls (blue) to essential tremor patients (green). Individual data points are represented by dots. For cognitive scores, higher values reflect better cognitive performance, while higher clinical scores indicate worse symptoms in patients. ET = essential tremor, RMET = reading the mind in the eyes test, FPRT = Faux Pas Recognition Test.

Patients (N = 21) did not significantly differ from controls (N = 29) with respect to sex distribution (*p* = 0.575), age (*p* = 0.335), or education level (*p* = 0.600; see also Table and Fig. 1). The disease duration in the ET group was 13.9 ± 12.3 years with a TETRAS mean of 30.7 ± 18.5 points. *General Cognition*: The control group showed higher scores in the PANDA test used for cognitive screening test (*p* = 0.004, r = 0.40). *Executive Functioning*: The patient group performed significantly worse than the control group in Verbal Fluency tasks (*p* = 0.001, d = 0.99) and Digit Span tasks (*p* = 0.006, d = 0.82). *Theory of Mind*: The patient group performed significantly worse than the control group in the FPRT (*p* = 0.003, r = 0.41). The RMET performance did not differ significantly between the groups, but a trend to worse performance in the patients compared to controls was seen (*p* = 0.052, d = 0.57). *Clinical Scales*: According to the Hamilton Depression Scale, mood was lower in patients than controls, but none of the values indicated a clinically relevant case of depression (*p* = 0.016, r = − 0.34). The PDQ-39 results also indicated worse health-related quality of life in patients compared to controls (*p* = 0.006, r = − 0.40). No significant differences were found in Sleepiness (*p* = 0.341) and Fatigue (*p* = 0.869). For an overview see Table 1.

**Table 1:** ET = Essential tremor, N = Total participants, n = Number of participants (e.g., sex), mean ± SD = Mean and standard deviation, U = Mann–Whitney U statistic, t(df) = t-statistic (degrees of freedom), d = Cohen’s d (effect size for t-test), r = Effect size for Mann–Whitney U test, RMET = Reading the Mind in the Eyes Test (cognitive empathy). Bold p-values mark significant differences.

Characteristic	Controls (mean ± SD)	ET patients (mean ± SD)	Test statistics	*p*-value	Effect size
	N = 29	N = 21			
Sex (*female*)	n = 12	n = 7	U = 255.00	0.575	
Age (*years*)	63.21 ± 13.60	63.67 ± 20.12	U = 329.00	0.335	
Education (*years*)	11.34 ± 1.88	11.53 ± 2.16	U = 315.50	0.600	
TETRAS		30.70 ± 18.5			
ET duration (years)		13.9 ± 12.3			
General cognition	26.72 ± 3.97	24.14 ± 3.65	U = 449.00	0.004	r = 0.40
Verbal fluency	83.03 ± 19.99	63.48 ± 19.28	t(48) = 3.47	0.001	d = 0.99
Digit span	17.55 ± 4.72	13.52 ± 5.11	t(48) = 2.88	0.006	d = 0.82
RMET	24.41 ± 4.29	21.67 ± 5.44	t(48) = 2.00	0.052	d = 0.57
Faux pax	0.95 ± 0.08	0.84 ± 0.13	U = 452.50	0.003	r = 0.41
Depression	2.45 ± 2.41	5.05 ± 4.24	U = 182.50	0.016	r = -0.34
Quality of life	6.98 ± 9.26	18.45 ± 15.02	U = 131.00	0.006	r = -0.40
Sleepiness	7.14 ± 5.01	5.95 ± 4.62	U = 321.00	0.341	r = 0.14
Fatigue	23.83 ± 8.87	24.50 ± 12.15	U = 269.00	0.869	r = 0.03

### Linear mixed models

In the verbal fluency mixed linear model, ET patients performed significantly worse than healthy controls (z = − 3.28, *p* = 0.001). While non-alternating tasks showed no significant main effect (z = 1.27, *p* = 0.203), a significant interaction between non-alternating and semantic verbal fluency tasks was observed (z = − 5.18, *p* < 0.001). No other interactions between group, task type, or condition reached statistical significance.

In the digit span mixed linear model, ET patients showed significantly lower performance (z = − 3.16, *p* = 0.002). Forward digit span tasks were associated with a significant improvement in performance across both groups (z = 3.20, *p* = 0.001). No interaction was identified between group and condition (z = 1.30, *p* = 0.192).

### Cognition vs psychometric functioning

*Control Group.* The general cognitive state screened by the PANDA was strongly correlated with verbal fluency (r = 0.60, *p* < 0.001) and moderately with digit span performance (r = 0.45, *p* = 0.014). Verbal fluency correlated with digit span performance (r = 0.55, *p* = 0.001), RMET (r = 0.52, *p* = 0.003), and FPRT (r = 0.42, *p* = 0.025), while being inversely related to life quality (r = − 0.49, *p* = 0.009). Digit span correlated positively with RMET (r = 0.53, *p* = 0.003) and negatively with quality of life (r = − 0.40, *p* = 0.036). Additionally, depression was positively correlated with sleepiness (r = 0.38, *p* = 0.040). Other relationships did not reach statistical significance.

*ET Patient Group*. In the ET group, general cognition was positively associated with verbal fluency (r = 0.60, *p* = 0.003) and verbal fluency correlated with digit span (r = 0.75, *p* < 0.001) and RMET performances (r = 0.68, *p* < 0.001). Further, the digit span results were linked to RMET (r = 0.55, *p* < 0.01) and FPRT performances (r = 0.57, *p* < 0.01). Depression and quality of life were associated with fatigue (r = 0.53, *p* = 0.022/r = 0.60, *p* = 0.008). Disease duration correlated with cognitive screening results on a trend level (r = 0.39, *p* = 0.083). Other correlations between variables were not identified.

## Discussion

The current data indicates that previously shown subtle cognitive dysfunctions in patients with ET extend to the domain of social cognition. Specifically, in comparison to control persons, subjects with ET scored abnormally low in the cognitive screening, verbal fluency, and digit-span tests as well as in cognitive ToM performance measured by the FPRT. Affective ToM performance assessed by the RMET was not significantly different between the groups. With respect to relevant clinical parameters, health-related quality of life was significantly lowered and the score for depressiveness increased. No differences were identified with respect to fatigue or daytime sleepiness.

Generally, the current findings tie in with the assumption of a leading dysexecutive deficit profile in ET^26,40^. Indeed, tasks in which the current ET patients performed abnormally low all implied relatively high demands of executive processing. For example, the tested verbal fluency performance requires access to and retrieval of words, the inhibition of wrong and already named, short-term memorized words, as well as switching between task conditions^41,42^. Accordingly, low verbal fluency is typically found in conditions with a dysexecutive cognitive profile, for example, in Parkinson’s disease^43,44^. The digit span tasks focus on working memory, the reduction of which was proposed to be mediated by a genuine dysexecutive problem in ET^26^. For example, the backwards condition requires complex reconfiguration operations for the memorized sequences, necessitating the inhibition of the primarily remembered order in favor of the release of its inverted version. Interestingly, the additionally identified performance reduction in the FPRT may be considered as a further facet within this framework. Associations of cognitive ToM and executive decline were reported previously in other scenarios, e.g., with respect to aging-related mental alterations^10,45–47^, but also in clinical conditions such as cervical dystonia or dysimmune neuropathy^17,18^. A reason for this link could be that cognitive ToM—and so FPRT—performance implies executive and working memory operations, i.e., switching between the perspectives of different protagonists interacting with each other, selecting information, updating it during its evolution, and so on^11,25^. Thus, social cognitive deficits could, just as working memory problems, be mediated by underlying executive dysfunctions in ET. Supporting this view, in patients and controls different combinations of verbal fluency and digit span results on the one hand and the social cognitive test performances on the other hand were correlated with each other. The origin of executive and working memory deficits in ET is not clear, but an interesting proposal is that they arise from the dysfunction of large-scale cerebellar-thalamic-prefronal/parietal networks^48^. In this view, the ET-related brainstem and cerebellar pathology eventually impacts on cortical processing and, thus, mediates the cognitive symptoms, which characterize ET beyond the eponymous tremor^40^. Tying in with this concept, executive dysfunction with a marked decrease of verbal fluency was shown in different cerebellar pathologies^49,50^ and a link between social cognitive behaviors and cerebellar function was suggested based on fMRI studies^51,52^.

With respect to affective ToM, it deserves mention that even if a significant RMET difference between the groups was absent, a statistical trend to lower test performance in the ET group was present. In this regard, the relatively small cohorts may have favored the missing of minor group differences. Thus, the interpretation of the RMET data remains ambiguous. In favor of an exclusive cognitive ToM deficit in ET, one might argue that executive demands are not only implied in the FPRT, but, to a lesser extent, also in the RMET (e.g., for selecting adjectives matching the pictured expression). Accordingly, executive dysfunction in ET could underlie reduced RMET performance, which could also explain the significant correlations with the abnormally low results in the verbal fluency and digit span tests. Alternatively, it is conceivable that ET-related disturbance of an affective ToM network implying the cerebellum facilitated low RMET performance, given connectivity analyses suggesting cerebro-cerebellar mentalizing functions or observations of emotion recognition problems in degenerative or ischemic cerebellar diseases^51–55^. In sum, larger studies would help to scrutinize whether affective ToM decline is restricted to the cognitive domain or if it also comprises its affective aspects. In so doing, also further open topics relevant in the context of social cognition could be addressed, e.g., concerning potential age or gender-related modulation of executive and ToM (dys)functions^56–58^. Finally, it should be considered that the measured affective state was significantly worse in the ET group than in controls. Thus, it cannot be ruled out that depressiveness contributed to disease-related reductions in cognitive performances. Against this possibility speaks the only slight elevation of Hamilton Depression Scale values and their absent correlation with cognitive task performances. However, even if depressiveness facilitated low cognitive performances in the ET group, this would be a genuine phenomenon in ET, since a lowered affective state is a regular non-sensorimotor aspect of the disease^59^.

In conclusion, ET appears to imply deficits in executive capacities, also affecting social cognitive performance. This extends previous reports on mental symptoms in this condition. Clinically, the results call for a closer view on the non-motor symptoms of patients with ET and their daily life impact.

## Data Availability

The datasets used and/or analysed during the current study are available from the corresponding author on reasonable request.
